# Unmet supportive care needs of breast cancer survivors: a systematic scoping review

**DOI:** 10.1186/s12885-023-11087-8

**Published:** 2023-06-26

**Authors:** Rongrong Fan, Lili Wang, Xiaofan Bu, Wenxiu Wang, Jing Zhu

**Affiliations:** 1 Department of Pulmonary and Critical Care Medicine, West China Hospital, Sichuan University/West China School of Nursing, Sichuan University, No.37, Guoxue Lane, Wuhou District, Chengdu, Sichuan China; 2 The School of Nursing, The Hong Kong Polytechnic University, Hung Hom, Kowloon, Hong Kong

**Keywords:** Breast neoplasms, Unmet supportive care needs, Systematic scoping review, Influencing factors, Breast cancer survivors

## Abstract

**Background:**

Breast cancer is the most common type of cancer in women worldwide. Though improved treatments and prolonged overall survival, breast cancer survivors (BCSs) persistently suffer from various unmet supportive care needs (USCNs) throughout the disease. This scoping review aims to synthesize current literature regarding USCNs among BCSs.

**Methods:**

This study followed a scoping review framework. Articles were retrieved from Cochrane Library, PubMed, Embase, Web of Science, and Medline from inception through June 2023, as well as reference lists of relevant literature. Peer-reviewed journal articles were included if USCNs among BCSs were reported. Inclusion/exclusion criteria were adopted to screen articles’ titles and abstracts as well as to entirely assess any potentially pertinent records by two independent researchers. Methodological quality was independently appraised following Joanna Briggs Institute (JBI) critical appraisal tools. Content analytic approach and meta-analysis were performed for qualitative and quantitative studies respectively. Results were reported according to the PRISMA extension for scoping reviews.

**Results:**

A total of 10,574 records were retrieved and 77 studies were included finally. The overall risk of bias was low to moderate. The self-made questionnaire was the most used instrument, followed by The Short-form Supportive Care Needs Survey questionnaire (SCNS-SF34). A total of 16 domains of USCNs were finally identified. Social support (74%), daily activity (54%), sexual/intimacy (52%), fear of cancer recurrence/ spreading (50%), and information support (45%) were the top unmet supportive care needs. Information needs and psychological/emotional needs appeared most frequently. The USCNs was found to be significantly associated with demographic factors, disease factors, and psychological factors.

**Conclusion:**

BCSs are experiencing a large number of USCNs in fearing of cancer recurrence, daily activity, sexual/intimacy, psychology and information, with proportions ranging from 45% to 74%. Substantial heterogeneity in study populations and assessment tools was observed. There is a need for further research to identify a standard evaluation tool targeted to USCNs on BCSs. Effective interventions based on guidelines should be formulated and conducted to decrease USCNs among BCSs in the future.

## Highlights

- A total of 16 domains of USCNs were finally identified.
- Social support, fear of cancer recurrence, and daily activity were the top unmet supportive care needs among breast cancer survivors. Information needs and psychological needs were reported frequently.
- Unmet supportive care needs were significantly affected by demographic factors, disease factors, and psychological factors.
- Substantial heterogeneity in study populations and assessment was observed. Assessment tools that specifically to unmet supportive care needs in breast cancer survivors were absent.

## Introduction

Breast cancer is a global cause for concern owing to its high incidence among women around the world [1]. According to the Global Cancer Statistics 2020 [2], female breast cancer has surpassed lung cancer as the most commonly diagnosed cancer, with an estimated 2.3 million new cases (11.7%). With improvements in early detection, surgery, and adjuvant therapy for breast cancer, long-term survival and cure are becoming possible. It is estimated that currently, 5-year survival rates are in the range of 90%, and 10-year survival is about 80% [3]. Quality of life is thus becoming a major issue for these patients. Nevertheless, many of them continue to be burdened by psychological distress and poor quality of life throughout their cancer trajectory [4]. Postoperative complications and side effects of chemoradiotherapy leave serious impacts on multiple aspects of their life, resulting in fatigue, sleep disorder, limb dysfunction [5], and even severe psychological matters [6]. Some recent studies unveiled that BCSs has endorsed moderate to high levels of depressive symptoms, anxiety, and post-traumatic stress [7]. Therefore, they report increased supportive care needs that require high-quality care in the domains of psychosocial, informational, and relational perspective [8, 9].

Supportive care encompasses a person-centered approach to care that aims to help a person with cancer and their family to meet their needs at multiple levels, from pre-diagnosis through the process of diagnosis and treatment to cure, continuing illness or death and into bereavement [10, 11]. The term “supportive care needs” is an umbrella term covering the physical, informational, emotional, practical, social, and spiritual needs of a person affected by cancer [12].

To ensure patients’ needs are addressed, there has been an increasing interest in supportive care needs assessment. Needs that were not well addressed and where additional support was required were classified as ‘unmet needs’ [11]. There is a growing body of literature that recognizes the significance of unmet supportive care needs (USCNs) among BCSs [13–15]. In the healthcare field, USCN reflects incongruity between the supports that an individual perceives to be necessary versus the actual supports provided [16]. It can be seen as covering a spectrum of healthcare needs that are not optimally met [17]. USCNs assessment is a patient‐oriented approach, which can lead resources to be distributed efficiently, and bring better outcomes for patients as finite medical resources could be directed to the benefit of patients with the greatest needs [18]. The ultimate goal is increasingly aligned and predictable pathway for the management and assessment, to meet the most required supportive care needs.

There is increasing evidence that USCNs can have a detrimental effect on BCSs’ well-being [19]. Accurate identification of USCNs of BCSs not only increases their satisfaction, but also improve their quality of life [20, 21]. Nevertheless, the knowledge about the most primary USCNs breast cancer patients are facing remains inadequate and unclear. Systematic reviews regarding USCNs were performed in some cancer groups, such as advanced cancer patients and their caregivers [22], prostate cancer patients [23], lung cancer [24], bladder cancer [25], and head and neck cancer [26]. Despite much observational study has been conducted, limited research has focused on any systematic review into USCNs among BCSs. A comprehensive understanding of USCNs among BCSs is crucial to direct future research and clinical practice. Therefore, a cohesive and up-to-date synthesis of the literature is needed to describe the USCNs of BCSs, which can inform the design and delivery of quality supportive care for this growing and diverse subpopulation, as well as guiding thinking to shape effective, evidence-based interventions. The main objective of this systematic scoping review is to identify, analyze and synthesize existing literature regarding the USCNs among BCSs and organize them into a structure from which the reader can obtain an in-depth understanding of this topic.

## Methods

### Review framework

This study employed a scoping review methodology to examine the range and scope of the available literature on the investigated topic, producing a rigorous synthesis and disseminating the existing evidence to date. The scoping review followed a methodological framework including the following five-stage process [27]: identifying the research question; identifying relevant studies; study selection; charting the data; and collating, summarizing, and reporting the results.

This review was conducted following the Preferred Reporting Items for Systematic Reviews and Meta-Analyses guidelines Extension for Scoping Reviews (PRISMA-ScR) [28]. The protocol was registered in PROSPERO with a registration number of CRD42022360528.

### Review questions

1. What are the USCNs of BCSs?
2. How many categories of domains of USCNs can be divided?
3. Which USCNs accounts the most proportion among BCSs?
4. What are the factors that might influence the USCNs?

### Search strategy

An extensive search strategy was conducted in Cochrane Library, PubMed, Embase, Web of Science, and Medline from inception through June 2023. Medical subject headings (MeSH) and text words were used to identify studies. The search strategy for ‘unmet supportive care need’ was Search #1: “needs assessment” [MeSH Terms] OR “needs assessment” [Title/Abstract] OR “assessment of healthcare needs” [Title/Abstract] OR “assessment of health care needs” [Title/ Abstract] OR “unmet needs” [Title/Abstract] OR “supportive care” [Title/Abstract] OR “need” [Title/Abstract]. The search strategy for ‘breast cancer survivor’ was Search #2: "breast neoplasms"[MeSH Terms] OR “breast neoplasms” [Title/Abstract] OR “breast cancer” [Title/Abstract] OR “breast tumor” [Title/Abstract] OR “breast oncology” [Title/Abstract]. An extended range search was carried out through ‘Search #1’ And ‘Search #2’. Furthermore, a snowballing strategy was also used with reference lists of relevant literature to locate additional studies not identified in the search strategies.

### Eligibility criteria

#### Participants criteria

According to the definition of the National Cancer Institute (NCI), survivor signifies one who remains alive and continues to function during and after overcoming a serious hardship or life-threatening disease. In cancer, a person is considered to be a survivor from the time of diagnosis until the end of life. It can be extended that breast cancer survivors refer to breast cancer individuals from the time of breast cancer diagnosis through the process of their lifespan. Thus, breast cancer survivors and breast cancer patients were both regarded as survivors in the present study. The criteria for participants were determined based on this premise: adult survivors (≥ 18 years) who were diagnosed with breast cancer, regardless of cancer stage, and current treatment, were eligible.

#### Studies

Studies investigating USCNs of BCSs were included. The eligibility criteria for selecting studies are listed as follows:

#### Inclusion criteria

• Any study published in a peer-reviewed journal of qualitative or quantitative design.
• English articles were included only to obtain articles with enough authoritativeness and professionalism, as well as to avoid language barriers and translation bias.
• USCNs were reported as primary or secondary outcomes (or expressed in terms of an unresolved desire for support/service provision/concerns that are explicitly referred to and measured as ‘unmet needs’).

#### Exclusion Criteria

• Conference articles, abstracts, editorial comments, guidelines, or unpublished works.
• Any study that included a mixed population, the results were reported together and could not be separated for breast cancer.
• The reported outcome from patients in the terminal or end-of-life care phase (final weeks/days of life).
• Any study solely focused on the presence of quality of life, satisfaction, or some specific unmet need (such as unmet symptoms/ psychology problems/ reproductive concerns/ rehabilitation/ diet and so on).

### Quality assessment

For each included study, methodological quality was independently appraised by two authors following Joanna Briggs Institute (JBI) critical appraisal checklist, which was recommended for studies reporting prevalence data and also suitable for qualitative studies [29]. It aims to assess the methodological quality of studies and to determine the extent to which a study has addressed the possibility of bias in its design, conduct, and analysis. When disagreement occurred, a consensus was reached by discussion. The JBI critical appraisal checklist for qualitative research and prevalence research could be divided into 10 and 9 measurement properties, respectively. As for mixed studies, we used both tools for each part. For the qualitative part, JBI critical appraisal checklist for qualitative research was used. For the quantitative part, JBI critical appraisal checklist for studies reporting prevalence data was applied. Each question option can be rated as “yes”, “no”, “unclear”, or “not applicable”. In each item, the percentage of each option was calculated and multiplied by 100%. The higher ‘yes’ responses on the appraisal items indicated a study of superior quality. The risk of bias scores was categorized based on “yes” rates as ≥ 80% (low), 60 to 80% (moderate), and < 60% (high).

### Study selection and data extraction

Two independent researchers performed double-checks on literature screening and data extracting. In an initial round of screening, study authors reviewed the titles and abstracts in the consolidated dataset for relevance based on the abovementioned inclusion/exclusion criteria. In a secondary screening, articles were reviewed in their entirety and incorporated into the present review if they met the eligibility criteria. Disagreements were addressed via frequent discussions with a third independent author or between the authors. A final set of articles fitting the scope of the present review were analyzed and summarized. A pre-defined Excel form was formulated specifically for this review to facilitate the extraction of pertinent data. The columns of the characteristics of the included studies were designed and the key information relevant to the review question were recorded. Essential information was extracted from eligible articles involving title, authors, country of origin, year of publication, sample size, population demographics, research design, assessment tools, main finding, the proportion of unmet needs, and factors related to USCNs. Whereby studies measured USCNs at multiple time points, all data corresponding to the different time points were extracted. However, only baseline measures were used for data synthesis in tables and figures.

### Data analysis and synthesis

For qualitative studies, the content analytic approach was applied to narrative synthesis. For quantitative studies that reported the prevalence of USCNs, total participants, domain categories and proportion were recorded and calculated. If there was any study that reported two or more USCNs with varying proportions in a given domain, the median proportion was calculated (i.e., if a study reported multiple items in the domain of unmet psychological need, such as stress, anxiety, and depression with different proportions, the median proportion was calculated to represent the whole rate of the domain). The larger median proportion indicates a higher USCN.

The meta-analysis was performed using Review Manager Software (version 5.3). The pooled proportions (with respective 95% CIs) for each domain were calculated. To explore heterogeneity between the studies the I^2^ statistics were used. Given the heterogeneity of estimates, a random-effects model was set. When I^2^ was > 0.50% the statistical heterogeneity was considered substantial. We limited meta-analysis to quantitative studies that applied comprehensive (multiple domains) needs assessments: This was to ensure some comparability between pooled studies, and to avoid inflation of estimates that may arise from targeted assessment in a single domain. Tables and bar charts will be used to present the main results.

## Results

### Literature search

**Fig. 1:**
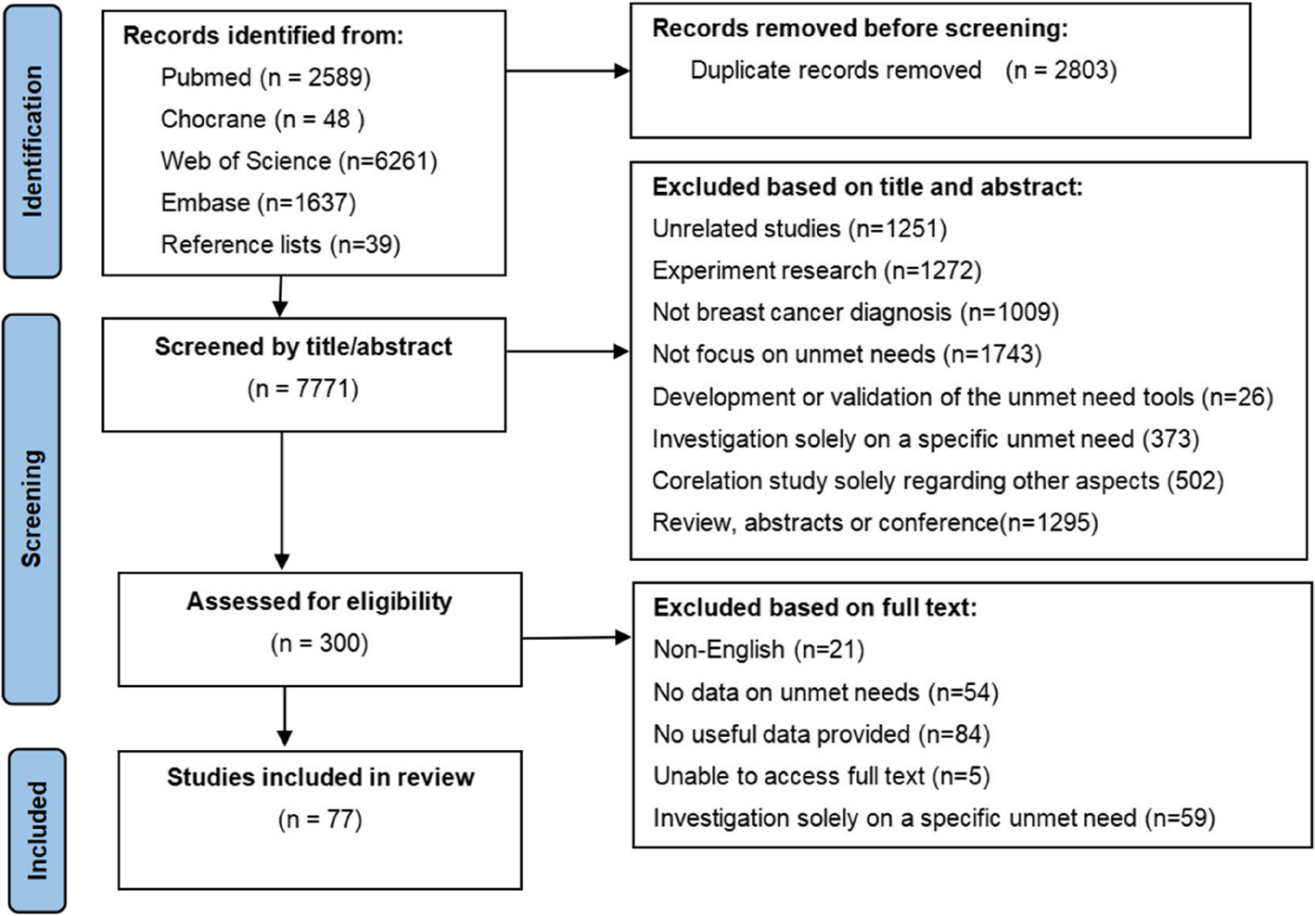
PRISMA diagram of systematic search and selection procedure

A total of 10,574 records were retrieved. After excluding 2803 duplicates, a total of 7771 studies were retrieved for titles and abstracts screening. After screening for titles and abstracts, 7471 articles were excluded and 300 papers were retrieved for full-text review. The final 77 articles were included, which consisted of 21 qualitative studies, 52 quantitative studies, and 4 mixed studies. The flow chart of the literature search is shown in Fig. 1.

### Quality assessment

**Fig. 2:**
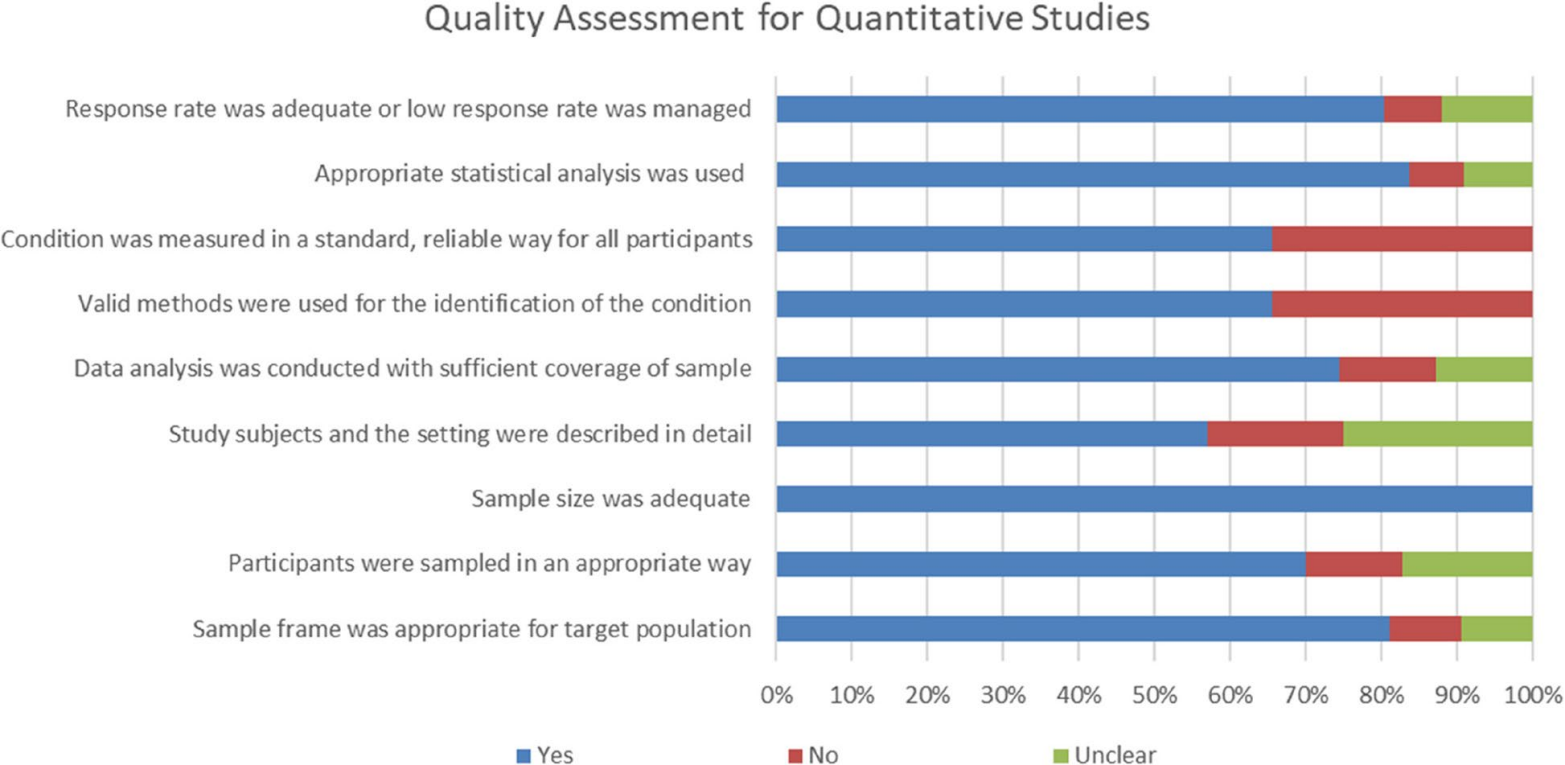
Quality assessment for quantitative studies

**Fig. 3:**
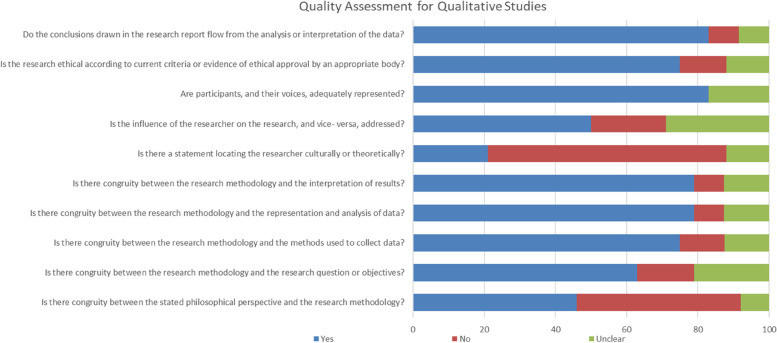
Quality assessment for qualitative studies

The overall risk of bias is shown in Figs. 2 and 3. More than 6o% of the quantitative studies had ‘Yes’ responses to all nine items. Nearly 34.5% had ‘No’ responses to the “Condition was measured in a standard, reliable way for all participants” item and “Valid methods were used for the identification of the condition” item. A few studies had “Unclear” responses on the “Study subjects and the setting were described in detail” item (about 25.6%). Among qualitative studies, nearly 60% of articles had “No” responses to the “Is there a statement locating the researcher culturally or theoretically?” item. Nearly 29% of articles had “Unclear” responses to the “Is the influence of the researcher on the research, and vice-versa, addressed?” item, and 67% had “No” responses to the “Is there a statement locating the researcher culturally or theoretically?” item.

### Literature characteristics

The final 77 articles were included, which consisted of 52 quantitative studies, 21 qualitative studies, and 4 mixed studies [30–32]. For mixed studies, the quantitative part was assigned as the quantitative study, and qualitative part was assigned as the qualitative study. Therefore, there are 56 quantitative studies and 25 qualitative studies that were included in the final analysis. The literature characteristics were summarized in Table 1. The publication period is from 2004 to 2023. There were 33 (42.9%) studies that are published after 2018. The United States, China, Korea, Australia, and the UK published the most articles. Most quantitative studies were cross-sectional design. The most used instrument was the self-made questionnaire (19, 33.9%) [33–50], followed by The Short-form Supportive Care Needs Survey questionnaire (SCNS-SF34) (12, 23.2%) [31, 51–63], Supportive Care Needs Survey (5, 8.9%) (SCNS) [64–68], Cancer Survivors Unmet Needs (3, 5.4%) (CaSUN) [19, 69, 70] and The Comprehensive Needs Assessment Tool (2, 3.6%) (CNAT) [30, 71]. In-depth, semi-structured interview was the most used approach in qualitative studies. The majority of the participants included in this review were women diagnosed with breast cancer who were in the post-treatment period. Only five studies involved objects who were undergoing treatment. There were 16 domains of USCN were finally identified, they were: physical/symptom need, psychological/emotional need, fear of cancer recurrence/ spreading, family support, medical support, social support, financial support, sexual/intimacy need, coping/survival need, daily activity need, spiritual support, information support, medical counseling, peer communication, cognitive needs, and dignity.

**Table 1:** Literature characteristics

Author team	Year	Country	Study Design	Supportive Care Needs Assessment Tool	Number	Participant	Age	Domains
Baker et al. [33]	2019	UK	Cross-sectional	SMQ	980	BCSs during or after cancer treatment	50–54	PS: 67.2%, PE: 77.6%
Vuksanovic et al. [72]	2021	Australia	Cross-sectional	CSUNQ	130	BCSs diagnosed at least one year	NR	PS: 29.1%, PE: 31.6%, FCR: 41.1%, Inf: 26.1%, MC: 29.9%
Abdollahzadeh et al. [64]	2014	Iran	Cross-sectional	SCNS	136	BCSs who finished the initial treatment	46.8 ± 10.1	PS: 67.8%, PE: 62.7%, FS: 60.5%, Sex: 59.1%, Act: 67.8%, Inf: 70.7%
Akechi et al. [51]	2011	Japan	Cross-sectional	SCNS-SF34	408	BCSs at all stages and at any time point after diagnosis	56.1 ± 12.1	PE: 48%, FCR: 63%, Inf: 45.5%, MC: 50%
Autade et al. [34]	2021	India	Cross-sectional	SMQ	120	BCSs at any stage and have completed primary treatment	52	PS: 100%, PE: 100%, FS: 40%, Cop: 32.5%, SP: 40%
Barr et al. [15]	2020	Victoria	Cross-sectional	SCNS-Breast	202	Young BCSs in early survivorship diagnosed with stage I or stage II	43.5 ± 5.0	PE: 67.5%, Act: 63%, Inf: 64%, MC: 64%, PC: 44%
Batehup et al. [19]	2021	UK	Cross-sectional	CaSUN	540	BCSs in the first 8 months post-primary treatment	61.2 ± 11.6	FS: 85.2%, MS: 85%, SS: 90.9%, Sex: 86.3%, SP: 92%, Inf: 89.2%, Cog: 82.1%, PC: 87%
Bu et al. [73]	2022	Chinese Mainland	Cross-sectional	CSP-BC	1210	BCSs who had completed primary therapy	NR	FCR:69%, MS:49.7%, SS: 52%, Fns:48.5%, Act:53.1%, Inf:54.3%, MC:63.2%, Dig:59.5%
Burris et al. [69]	2015	USA	Cross-sectional	CaSUN	90	BCSs at stage I-III and had plans for radiation therapy	55.26	PS: 25.3%, PE: 27.6%, FCR: 31%, Fns: 28.7%, Cop: 31%, Inf: 36.7%, MC: 36.8%
Capelan et al. [74]	2017	UK	Cross-sectional	HNA + EPR	625	BCSs at the early stage(I–III) who had completed initial treatment	59 ± 13	PS: 55%, PE: 24%, FS: 5%, Cop: 6%, SP: 4%
Cheng et al. [52]	2014	Singapore	Cross-sectional	SCNS-SF34	150	BCSs at six months to five years post-treatment period	55.1 ± 8	PS: 44%, PE: 29%, Inf: 37%
Choi et al. [65]	2013	Chinese Mainland	Cross-sectional	SCNS	163	BCSs who completed first-line cancer treatment	NR	Inf: 59%
Chou et al. [75]	2022	Taiwan China	Cross-sectional	Records	1129	BCSs who were receiving treatment	46–55	PS: 3.5%, PE: 40.4%, MC: 11.9%, MS: 24.6%, Fns: 0.2%
Chua et al. [76]	2020	Singapore	Cross-sectional	MCCC-CSSN	438	BCSs	56 (25–81)	PS: 46.2%, FCR: 55%, MS: 37.4%
Chyon et al. [53]	2016	Korea	Cross-sectional	SCNS-SF34	117	BCSs before adjuvant therapy	45.1 ± 7.25	PS: 51.7%, PE: 57.7%, FCR: 79.5%, MC: 51.7%, Cop: 49.6%, Act: 52.7%, Inf: 65%
de Ligt et al. [35]	2019	Netherlands	Cross-sectional	SMQ	404	BCSs at early-stage during treatment	62 ± 10.9	PS: 63.4%, PE: 53%
Dugan et al. [36]	2021	USA	Cross-sectional	SMQ	76	BCSs with completed active primary treatment within the past 36 months	52.6 ± 10.7	PE: 22%, MS: 9%, Cop: 7%, Inf: 30%
Edib et al. [54]	2016	Malaysia	Cross-sectional	SCNS-SF34	117	BCSs at all ages and any stages and had survived at least one year after diagnosis	38.2 ± 27.2	PS: 56.5%, PE: 66.7%, FCR: 76.1%, Sex: 35%, Cop: 58.1%, Inf: 45.3%
Farrelly et al. [77]	2013	Australia	Cross-sectional	SMQ	279	BCSs who had been identified as carrying a BRCA1/2 mutation	46 ± 13.9	PE:32.9%, FCR:41.3%, FS:33.1%, Fns: 22.3%, Cop: 39.7%, Inf: 29.1%, PC: 35.5%
Fong et al. [55]	2016	Malaysia	Cross-sectional	SCNS-SF34	101	BCSs	57.9 ± 9.53	FCR: 16.8%, MS: 14.9%, Inf: 20.8%, MC: 3.2%
Shih^a^ et al. [78]	2020	Hong Kong China	Cross-sectional	CCSUNS	157	BCSs with survival duration 2–5 years	55.2 ± 10.6	PS: 49.7%, PE: 20.4%, FCR: 60.5%, Cop: 29.9%, Inf: 52.9%, MC: 25.8%, Dig: 21%
Shih^a^ et al. [78]	2020	Hong Kong China	Cross-sectional	CCSUNS	192	BCSs with survive duration over 5 years	57.34 ± 9.6	PS:18.2%, PE:15.1%, FCR:47.7%, FS:15.6%, MS:19%, Cop:10.4%, Inf:44.8%, MC:44.3%
Hwang et al. [66]	2006	Korea	Cross-sectional	SCNS	459	BCSs	NR	PE: 46.5%, MS: 53.8%, Inf: 48.8%, MC: 46.8%
Lam^b^ et al. [56]	2011	Hong Kong China	Cross-sectional	SCNS-SF34	348	Chinese BCSs	NR	PS: 10.6%, PE: 16%, FCR:16.4%, MS: 31.2%, Inf: 52%, MC: 52.2%
Lam^b^ et al. [56]	2011	Hong Kong China	Cross-sectional	SCNS-SF34	293	German Caucasian BCSs	NR	PS: 48.9%, PE: 43.6%, FCR: 57.1%, MS: 32%, Inf: 37%, MC: 35.6%
Garry^c^ et al. [37]	2013	UK	Mixed	SMQ	101	BCSs who were currently diagnosed or attending follow-up clinics	NR	PS: 44.5%, PE: 35%, MS: 65%
Meer et al. [38]	2017	British Columbia	Cross-sectional	SMQ	132	BCSs	NR	Act:58%, MC: 64%, FCR, Inf
Mirzaei et al. [57]	2019	NR	Cross-sectional	SCNS-SF34	190	BCSs under chemotherapy and radiotherapy	NR	PS: 14.5%, PE: 31.3%, Inf: 36%
Allison et al. [39]	2021	USA	Cross-sectional	SMQ	199	BCSs who had completed primary cancer therapy	59	PS: 55%, PE: 55%, FCR: 73%
Napoles et al. [79]	2016	Spanish	Cross-sectional	Tel-survey	118	BCSs with completed treatment within 10 years	NR	PS: 29.5%, PE: 33.7%, Act: 69%, Inf: 70%
Sleight et al. [80]	2018	USA	Cross-sectional	SCNS-SF34	99	BCSs with completed primary treatment	54.0 ± 8.6	PS:39%, PE:37.5%, FCR:49%, MS: 42%, Cop:35%, Act:49.5%, Inf:43%, MC:54%
Winnie et al. [58]	2014	Hong Kong China	Cross-sectional	SCNS-SF34	163	BCSs at one year after cancer treatment	NR	Inf: 59%
Wang^d^ et al. [59]	2018	Chinese Mainland	Cross-sectional	SCNS-SF34	121	Rural BCSs after treatment	49.5 ± 9.7	FCR: 57.8%, MS: 46.5%, Inf: 57%, MC: 49.2%
Wang^d^ et al. [59]	2018	Chinese Mainland	Cross-sectional	SCNS-SF34	143	Urban BCSs after treatment	49.5 ± 9.7	PE: 38.5%, FCR: 46.2%, MS: 36.4%, Cop: 47.6%, Act: 35.7%, Inf: 42%, MC: 44.1%
Annika et al. [40]	2013	Denmark	Cross-sectional	SMQ	261	BCSs during and after primary treatment for 4 months	60	Inf: 18%, MC: 15%, MS: 12%
Palmer et al. [41]	2017	NR	Cross-sectional	SMQ	103	BCSs diagnosed over 3 years	62.7	PS: 60%, Sex: 55%
Park et al. [67]	2012	Korea	Cross-sectional	SCNS	1084	BCSs at stages I, II, or III	NR	MS: 47.9%, Inf: 44%, MC: 43.7%
Park et al. [68]	2013	Korea	Cross-sectional	SCNS	52	BCSs	48.34 ± 8.3	PE:26.9%, FCR:33.1%, FS:29.6%, MS: 30.1%, Inf: 37.6%, MC:41.5%, PC: 29.6%
Silvia et al. [50]	2013	Switzerland	Cross-sectional	SMQ	175	BCSs under treatment	NR	PS: 79.6%, PE: 24.1%, Dig: 55.8%
Schmidt et al. [43]	2018	Germany	Cross-sectional	SMQ	190	BCSs survived 5 years after diagnosis	NR	PS: 37.5%, Cog: 36%
Tsung^e^ et al. [31]	2017	Malaysia	Mixed	SCNS-SF34	259	BCSs	56.2 ± 10.3	FCR:42.9%, Act
Ellegaard et al. [70]	2017	Denmark	Cross-sectional	CaSUN	155	BCSs between three months and five years after diagnosis	63	MS: 34.8%, Inf: 22.3%, Cop: 41.3%, PS: 20%, PE: 13.5, FCR: 16.1%
Hodgkinson et al. [45]	2007	Australia	Cross-sectional	SMQ	117	BCSs diagnosed 2–10 years	61	FCR:32.7%, Inf: 28.2%, MS: 21.8%, Cop:18.5%, Fns: 19.3%, MC: 18.5%
Winnie et al. [61]	2013	Hong Kong China	Cross-sectional	SCNS-SF34	376	BCSs completed treatment less than 1 year ago	53.8 ± 11.5	PS:7.2%, FCR:12%, PE:5.9%, Sex:3.7%, MS: 31.5%, MC: 19.1%, MS:35.9%, Inf: 30.7%
Elsous^g^ et al. [81]	2023	Palestine	Mix	SCNS-SF34	352	BCSs	NR	PE: 63%, Inf: 62%, PS:61%, Act: 61%
Chae et al. [71]	2019	Korea	Cross-sectional	CNAT	332	BCSs	NR	PS, FCR, MS, Inf, MC
Hernández et al. [60]	2019	Mexico	Cross-sectional	SCNS-SF34	186	BCSs during adjuvant endocrine therapy	54.5 ± 10.7	PS, PE, Fns, Sex, Dig
Han et al. [44]	2019	Korea	Cross-sectional	SMQ	146	BCSs who had undergone surgery and treatment	48.53 ± 8.2	PS, PE, MS, Inf, Sex
Lee et al. [30]	2021	Korea	Cross-sectional	CNAT	426	Physicians and BCSs	NR	PE, FCR, MS, Inf, MC
Burgmann et al. [82]	2016	Germany	Cross-sectional	QSCP	88	Young BCSs aged below 40	NR	FCR, Sex, PE, fear of further hospital stays
Chowdhury et al. [62]	2022	Bangladesh	Cross-sectional	SCNS-SF34	138	BCSs	40.5 ± 10.55	Inf
Fong et al. [63]	2019	Malaysia	Cross-sectional	SCNS-SF34	259	BCSs	56.2 ± 10.29	Fns, Cop, MS, FS, SS, SP
Gálvez et al. [83]	2018	Mexico	Cross-sectional	unmet SCN	150	Young BCSs	36	Inf
Gilmore et al. [46]	2014	USA	Cross-sectional	SMQ	114	Adult BCSs for their initial survivorship	NR	PS, PE, MS, Sex
Tan et al. [47]	2015	USA	Cross-sectional	SMQ	34	BCSs	64.7 ± 12.7	Cop, Inf
Wong et al. [48]	2020	USA	Cross-sectional	SMQ	746	BCSs in the first 15 months after diagnosis	NR	Act, MC
Thewes et al. [49]	2004	Australia	Cross-sectional	SMQ	95	BCSs	NR	PE, Inf, MC
Silvia et al. [50]	2011	Switzerland	Cross-sectional	SMQ	72	BCSs	57.5 ± 11.8	PS, PE, Sex
Cheng^f^ et al. [32]	2018	Singapore	Mixed	SCNS	250	BCSs with completed treatment	54.7 ± 8.2	MS, PE, PS, Inf, Sex
Elsous^g^ et al. [81]	2023	Palestine	Mixed	Interviews	25	BCSs	NR	MS, FS, SS, Sex, Dig
Cheng^f^ et al. [32]	2018	Singapore	Mixed	Interviews	80	BCSs with completed treatment	55.3 ± 7.6	MS, Inf, SS, FCR, Fns, Cop
Beatty et al. [84]	2008	Australia	Qualitative	Interviews	34	Early-stage BCSs within the past 12 months	53.5 ± 12.5	PS, Cog, PE, Cop
Adams et al. [85]	2017	USA	Qualitative	Interviews	15	Rural BCSs	NR	PS, Cog, PE, Cop, Inf, SP, SS
Dönmez et al. [86]	2021	Turkey	Qualitative	Interviews	19	BCSs with breast cancer-related lymphedema	52.15 ± 7.7	PS, Act, PE, SS, Inf, FS
Beaver et al. [87]	2016	UK	Qualitative	Interviews	20	BCSs with completed neo-adjuvant chemotherapy	NR	Inf, PE
Brown et al. [14]	2018	USA	Qualitative	Interviews	68	BCSs with gender minority	18–75	Cog, Sex, SS
Li et al. [88]	2014	Chinese Mainland	Qualitative	Interviews	154	BCSs who had undergone surgery	NR	Inf, PS, Sex, Dig
Cheng et al. [89]	2016	Chinese Mainland	Qualitative	Interviews	29	BCSs	NR	FCR, PS, Dig, Sex, Fns
Cheng et al. [32]	2017	Singapore	Qualitative	Interviews	60	BCSs	NR	Cop, MS, Act
Ddungu et al. [90]	2018	Uganda	Qualitative	Interviews	252	BCSs with metastatic breast cancer	NR	PS, Act, Inf, Cog, MS, PE, Cop, Dig
Dsouza et al. [91]	2018	India	Qualitative	Interviews	17	BCS	NR	Fns, Inf, Dig, Act, FS, PE, MC
Enzler et al. [92]	2019	USA	Qualitative	Interviews	37	BCSs received or receiving treatment	NR	Cop, Inf
Lindsey et al. [93]	2016	USA	Qualitative	Interviews	41	BCSs	NR	MS, Dig, SS, PE
Hubbeling et al. [94]	2018	USA	Qualitative	Interviews	25	Young BCSs	37–53	PE, Dig, SS, FS, Inf
Keesing et al. [95]	2019	Australia	Qualitative	Interviews	26	BCSs and partners	NR	PS, FCR, SS, Sex
Landmark et al. [96]	2008	Norway	Qualitative	Interviews	7	Newly diagnosed BCSs	NR	Inf, PE, SS
Garry^c^ et al. [37]	2013	UK	Mixed	Interviews	7	BCSs who were currently diagnosed or attending follow-up clinics	NR	MS
Oxlad et al. [97]	2008	Australia	Qualitative	Interviews	10	BCSs following primary treatment	36–68	PE, PS, Sex, FCR, Fns
Nápoles et al. [98]	2017	USA	Qualitative	Interviews	34	BCSs	NR	PS, SS, PE, MS, Inf, FCR
Tanjasiri et al. [99]	2011	USA	Qualitative	Interviews	20	BCSs	NR	Inf, SS, SP
Tsung^e^ et al. [31]	2017	Malaysia	Mixed	Interviews	9	BCSs	56.2 ± 10.3	Cop, SS, SP
Pembroke et al. [100]	2020	USA	Qualitative	Interviews	17	BCSs previously treated with radiation therapy	50	PE, Fns, MS, SS, Dig, Sex, Inf
Ruddy et al. [101]	2013	USA	Qualitative	Interviews	36	Young BCSs	18–42	Dig, Inf, PS, SS, Cop
Ruddy et al. [102]	2015	USA	Qualitative	Interviews	20	Young BCSs	> 42	Dig, FS

*BCSs* breast cancer survivors, *CSUNQ* Cancer Survivors Unmet Needs Questionnaire, *SCNS* Supportive Care Needs Survey, *SCNS-SF34* The Short-form Supportive Care Needs Survey questionnaire, *SCNS-Breast* Supportive Care Needs Survey-Breast Cancer, *CaSUN* Cancer Survivors Unmet Needs Survey, *CaSUN-S* Spanish Version of the Cancer Survivors' Unmet Needs, *CSP-BC* Cancer Survivor Profile-Breast Cancer, *HNA* Holistic Needs Assessment, *EPR* electronic patient record, *MCCC-CSSN* Mayo Clinic Cancer Centre's Cancer Survivors Survey of Needs, *CNAT* The Comprehensive Needs Assessment Tool, *SCN* The unmet supportive care needs, *SMNAI* Survivors Module Needs-Assessment Instrument, *CCSUNS* Chinese Cancer Survivors' Unmet Needs Scale, *QSCP* Questionnaire on Stress in Cancer Patients; unmet SCN: unmet supportive care needs, *SMQ* Self-made questionnaire, *PS* Physical/symptom, *PE* Psychological/emotional, *FCR* Fear of cancer recurrence/ spreading, *FS* Family support, *MS* Medical support, *SS* Social support, *Fns* Financial support, *Sex* sexual/intimacy, *Cop* Coping/survival, *Act* Daily activity, *SP* Spiritual support, *Inf* Information support, *MC* Medical counseling, *PC* Peers communication, *Cog* Cognitive needs, *Dig* Dignity, *NR* not clearly, a, b, d, different population in the same study (a, survived < 5 years vs survived > 5 years; b, Chinese vs German; d, rural population vs urban population); c, e, f, g: mixed study, the quantitative and qualitative sections were listed separately

### The estimated prevalence of USCNs from quantitative studies

The quantitative synthesis evaluating the proportion of USCNs in each domain were listed in Table 2. The most proportion of USCN was focused on social support (74%), daily activity (54%), sexual/intimacy (52%), fear of cancer recurrence/ spreading (50%), and information support (45%). However, the point estimate for social support should be interpreted with enough caution for they were extracted from two studies, which were highly inconsistent in their estimates [90.9% versus 52%]). The pooled estimate was based on a small sample, and the heterogeneity was large (I^2^ = 100%). There were amounts of studies that were excluded without the full text, which also may be one source of risk of bias.

**Table 2:** Estimated prevalence of USCNs by domains

Domain	No. of studies	Total N	Pooled proportion (%)	95% CI	I^2^ (%)
SS	2	1750	0.74	0.73,0.76	100
Act	11	2523	0.54	0.52,0.56	87
Sex	9	2556	0.52	0.52,0.56	99
FCR	24	5916	0.50	0.40,0.60	98
Inf	33	8352	0.45	0.37,0.52	98
PS	28	8346	0.43	0.32,0.54	100
PE	33	9814	0.42	0.33,0.5	100
Dig	4	1734	0.42	0.4,0.44	99
PC	3	533	0.38	0.34,0.42	60
MS	24	7803	0.36	0.27,0.44	99
Cog	4	2565	0.36	0.35,0.38	100
FS	9	2218	0.34	0.09,0.59	100
Cop	15	2521	0.34	0.24,0.44	98
MC	23	7223	0.33	0.32,0.34	99
SP	3	1285	0.32	0.3,0.33	100
Fns	5	2825	0.24	0.2,0.5	99

*PS* Physical/symptom, *PE* Psychological /emotional, *FCR* Fear of cancer recurrence/ spreading, *FS* Family support, *MS* Medical support, *SS* Social support, *Fns* Financial support, *Sex* sexual/intimacy, *Cop* Coping/survival, *Act* Daily activity, *SP* Spiritual support, *Inf* Information support, *MC* Medical counseling, *PC* Peers communication, *Cog* Cognitive needs, *Dig* Dignity

### Frequency of unmet needs

**Fig. 4:**
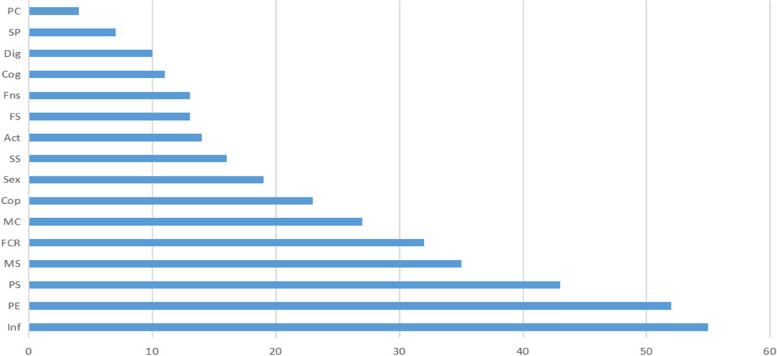
Frequency of unmet needs. PS: Physical/symptom; PE: Psychosocial/emotional; FCR: Fear of cancer recurrence/ spreading; FS: Family support; MS: Medical support; SS: Social support; Fns: Financial support; Sex: sexual/intimacy; Cop: Coping/survival; Act: Daily activity; SP: Spiritual support; Inf: Information support; MC: Medical counseling; PC: Peers communication; Cog: Cognitive needs; Dig: Dignity

By calculating the frequency of unmet domains (Fig. 4), information need (55) and psychological/emotional need (52) were been found to appear most frequently, followed by physical/symptom (43) medical support (35), and fear of cancer recurrence/ spreading (32).

### Prominent needs lists of each domain

The prominent needs with the median proportion of each domain were listed in Table 3. In physical/symptom domain, the frequently reported needs were lack of energy/tiredness [53.6% (10.6%-88.8%)], fatigue [51% (23%-87.7%)], pain [45.5% (18.5%-66%)], sleep disorder [44.9% (14%-57%)], and hot flashes [43% (23%-100%)]. In the psychosocial/emotional domain, the frequently reported needs were learning to feel in control of your situation [58.2% (47.9%-64.1%)], worrying that the results of treatment are beyond your control [54% (16.7%-71.8%)], concerns about the worries of those close to you [51.2% (43.4%-97.8%)], keep a positive outlook [49% (37%-53.8%)], and anxiety [48.7% (16%-90.6%)]. Fears of cancer spreading [57.5% (16.4%-80.3%)] and fear of cancer recurrence [47.9% (28.6%-73%)] play the predominant part in the fear of cancer recurrence/ spreading domain. Help to know how to support my family/ partner was the greatest USCN (85.2%) in family-related support. In the medical support field, the frequent USCNs were ongoing medical service [63%(37.4%-74.5%)], nutritional/diet needs [58%(28.4%-74)], wished to be able to obtain medical service in a quick and easy way when in need [50.9%(43.7%-85.5%)], reassurance by medical staff that the way you feel is normal [39.8% (30.8%-43%)], and hospital staff acknowledging, showing sensitivity to your feeling and emotion needs [38% (28.2%-48.8%)]. Help to handle the topic of cancer in social/work situations [53.5%(50.4%-90.9%)] was the highest USCN in social support. Diminished sexual activity/sexual drive was unveiled to be the prime unmet need in the interpersonal/intimacy/sexual support field. In survival/coping needs help to make new relationships (94%), dealing with my belief that nothing bad will happen again (85.2%), and dealing with the impact of cancer on my relationships (84.6%) were the prominent USCNs. Exercise need was the most mentioned in daily activity. Help with my spiritual beliefs counted 66%(40%-92%) in spiritual need. In health system/information, up to date understandable information about your cancer and treatment [62.5%(31.4%-89.5%)], being informed about cancer which is under control or diminishing (i.e., remission) [54.1%(20.8%-76.5%)], information related to hereditary of disease [52.5%(52.1%-52.9%)], and being informed about things you can do to help yourself to get well [51%(14.9%-80.9)] were the most pointed unmet needs. To have one member of the hospital staff with whom you can talk to about all aspects of your condition, treatment, and follow-up [45.5%(34.9%-87.7%)], spent time for discussing disease [45.3%(31.8%-63.2%)], and having access to professional counseling (e.g., psychologist, social worker, counselor, nurse specialist) if you, family, or friends need it [43.9%(27.7%-82%)] were mainly indicated in medical counseling. Talk to others who have been through a similar experience counted the most [40.4%(29.6%-87%)] in peers’ communication. Cognitive needs counted 37.8% [37.8%(36%-39.5%)]. Help to adjust to changes to the way I feel about my body (82.1%) was the primary issue in dignity needs.

**Table 3:** Prominent needs lists of each domain

Domain of needs	List	Median Proportion (min–max)
**Physical/** **symptom**	• Lack of energy/tiredness	53.6% (10.6%-88.8%)
	• Fatigue	51% (23%-87.7%)
	• Pain	45.5% (18.5%-66%)
	• Sleep disorder	44.9% (14%-57%)
	• Hot flashes	43% (23%-100%)
	• Osteoporosis/bone health	39% (37%-70.5%)
	• Numbness/tingling in hands/feet	35% (11%-41%)
	• Impairment of memory	33.1% (21%-48%)
	• Change in appetite	32.4%
	• Changes in weight	32% (10%-60%)
	• Dry: vaginal dryness, dry/itchy skin, dry nose/mouth	29% (11%-30%)
	• Manage side effects and complications of treatment	29.9% (3.5%-53.4%)
	• Constipation	24.3% (21.7%-26%)
	• Others: physical performance (39%), health problems regarding the breast (54%), reproductive system (58.2), urination changes (21%), and shortness of breath (21%)	
**Psychosocial/emotional**	• Learning to feel in control of your situation	58.2% (47.9%-64.1%)
	• Worry that the results of treatment are beyond your control	54.4% (16.7%-71.8%)
	• Concerns about the worries of those close to you	51.2% (43.4%-97.8%)
	• Keep a positive outlook	49% (37%-53.8%)
	• Anxiety	48.7% (16%-90.6%)
	• Feeling of uncertainty	46.2% (15.2%-92%)
	• Nervousness	44.6% (23%-66.1%)
	• Feeling down or depressed	44% (10%-82%)
	• Feelings about death and dying	42.2% (39%-68.4%)
	• Stress	35.6% (16.7%-77.5%)
	• Reassurance that the way you feel about your risk is normal	28.9%
	• Dealing with the loss of family members who had breast cancer	27.5%
	• Fears about physical disability or deterioration	26.9% (24%-42.4%)
	• Loss of interest in usual activities	24%
	• Dealing with feelings of isolation	22.4%
	• Emotional support	25% (15.1%-80.3%)
	• Changes to beliefs	4.5% (3.2%- 5.7%)
**Fear of cancer recurrence/ spreading**	• Fears cancer spreading	57.5% (16.4%-80.3%)
	• Fear of cancer recurrence	47.9% (28.6%-73%)
	• Dealing with the impact that having a faulty gene has had on your family	41.3%
	• Fear of further hospital stays	No data
**Family support**	• Help to know how to support my family/ partner	85.2%
	• Talking to other family members about having a faulty cancer protection gene	37.4%
	• Family or friends to be allowed with you in the hospital whenever you want	29.6%
	• Talking to your children about their cancer risk	28.8%
**Medical support**	• Ongoing medical service	63% (37.4%-74.5%)
	• Nutritional/diet needs	58% (28.4%-74%)
	• Wished to obtain medical service in a quick and easy way when in need	50.9% (43.7%-85.5%)
	• Reassurance by medical staff that the way you feel is normal	39.8% (30.8%-43%)
	• Hospital staff acknowledge, show sensitivity to your feeling and emotion needs	37% (28.2%-48.8%)
	• Hospital staff attending promptly to your physical needs	35.7% (27.3%-47%)
	• My doctors to talk to each other to coordinate my care	35.3% (9.6%-79.8%)
	• Being treated like a person not just another case	34.2% (25.6%-97.8%)
	• Feeling reassured that the best medical care is given	33.1% (9%-87.7%)
	• Being treated in a hospital(clinic) that is as physically pleasant as possible	32.9% (14.9%-41.9%)
	• To feel I can manage my health together with my health team	15.6% (8.9%-85%)
**Social support**	• Help to handle the topic of cancer in social/work situation	53.5% (50.4%-90.9%)
**Financial support**	• Financial strain/difficulties	26.2% (0.2%-48.5%)
	• Dealing with insurance issues that arise from having a faulty cancer protection gene	22.3%
**Sex/intimacy**	• Diminished sexual activity/sexual drive	70.7% (55%-86.3%)
	• Changes in sexual relationship	33.3% (19%-35%)
	• Change in sexual feeling	29% (25%-38.5%)
**Coping/survival**	• Help to make new relationships	94%
	• Help to deal with the impact of cancer on my relationships	84.6%
	• Help to make my life count	84.2%
	• Help to move on with my life	82.2%
	• Help to make decisions about my life in uncertain times	82.1%
	• Help to cope with others' expectations of me as a survivor	78.6%
	• Help with others not acknowledging the impact cancer has had on your life	60% (36.8%-83.2%)
	• Feeling unwell a lot of the time	51.3% (37%-97.8%)
	• Help to deal with my belief that nothing bad will happen again	41.5% (18.7%-87.2%)
	• Deciding how best to manage increased cancer risk	39.7%
	• Learning to feel in control of your situation	33%
	• Help manage household responsibility	31%
	• Adjust to changes in your life as a result of cancer	26.7%
	• Instrumental (practical) support	19.8(7%-32.5%)
**Daily activity**	• Exercise	69%
	• Physical activity to decrease the risk of recurrence or improve survival	55.6% (53.1%-63%)
	• Yoga/meditation	55%
	• Not being able to do the things you used to do	50% (29.1%-98.6%)
	• Work around the home	44.9% (39.3%-59.8%)
**Spiritual support**	• Help with my spiritual beliefs	42% (40%-92%)
**Information support**	• Up to date understandable information about your cancer and treatment	62.5% (31.4%-89.5%)
	• Being informed about cancer that is under control or diminishing (i.e., remission)	55.3% (20.8%-76.5%)
	• Information related to hereditary disease	52.5%(52.1%-52.9%)
	• Being informed about the things you can do to help yourself to get well	51% (14.9%-80.9)
	• Being given explanations on those tests about which you would like to get explanations	47% (29.7%-92%)
	• Being informed about your test results as soon as feasible	44.9% (20.8%-59.8%)
	• Being given information (written information, diagrams, and drawings) about aspects of managing your illness and side effects at home	44.2% (18.8%-73.5%)
	• Being given written information about important aspects of your care	44.2% (31.9%-97.1%)
	• Being adequately informed about the benefits and side effects of therapy before you choose to have them	41.5% (24.8%-91.3%)
	• Information resources	33.6% (28.7%-38.5%)
	• Information relevant to my partner/family	32.5% (28.1%-92.7%)
	• To be given choices about when to go in for tests or treatment	30.3%
	• Obtain information to help manage increased cancer risk	29.7%(29.1%-34.7%)
	• More choice about which cancer specialists you see	29.3% (19.3%-45.3%)
	• Be given information about sexual relationship	27.8% (19%-33.3%)
	• More choice about which hospital you attend	25.3% (21.4%-31.6%)
	• Patient education: diet:19%, relaxation/meditation: 18%, physical activity: 10%	
**Medical** **counseling**	• To have one member of the hospital staff with whom you can talk to about all aspects of your condition, treatment, and follow-up	45.5% (34.9%-87.7%)
	• Spent time discussing disease	45.3% (31.8%-63.2%)
	• Having access to professional counseling (e.g., psychologist, social worker, counselor, nurse specialist) if you, family, or friends need it	43.9% (27.7%-82%)
	• Spent time listening to feelings	31.5% (19.7%-43.2%)
	• Counselling: psychologist or psychiatrist: 15.5%(15%-16%), financial and occupational:15%	
**Peers communication**	• To talk to others who have been through a similar experience	40.4% (29.6%-87%)
	• Talking with other women who have faulty cancer protection gene	36%
	• Finding someone who understands your situation	32.3% (29.6%-35%)
**Cognitive needs**	• Cognitive needs	37.8% (36%-39.5%)
	• Memory or concentration problems	10%
**Dignity**	• Help to adjust to changes in the way I feel about my body	82.1%
	• Body image perception	38.4(8.9%-59.5%)

### Synthesis of unmet needs in qualitative studies

A content analytic approach was conducted to synthesize USCNs and categorize them into different domains. The result of the synthesis was listed in Table 4. In family support, participants not only expressed the need for support from family members but also presented the need in supporting their family members, which was in agreement with the result from quantitative research. In dignity, except for an unmet need in body image, more needs regarding disease disclosure were also expressed.

**Table 4:** Synthesis of unmet needs in qualitative studies

Domain of needs	Lists
Physical/symptom	Coping with side-effects [84, 97] Symptom management needs (pain, nutrition and diet, wound management, fatigue) [32, 85, 88–90, 95, 97]
Psychosocial/emotional	Stress and adjustment reactions [84], challenges resuming roles [98] Emotional support and empathy [32, 87] Sensitivity to feelings [90], sense of abandonment [98] Fertility concerns [94] Apprehension/uncertain/negativity about the future [86, 97], positive outlook [90]
Fear of cancer recurrence	Fear of recurrence [32, 89, 95, 97, 98]
Family support	Recognition and support from family/friends/partners [14, 91, 98] Lack of support services for cancer caregivers [93] Caregiver burnout [90] Appropriate support for their family and partners [14, 102]
Medical support	Attention from healthcare professionals [86] Continuity of care [32], the formal transition from active treatment to survivorship [98, 101] Pleasant environment, inadequate hospital amenities and medicines [90] Availability of anticancer therapy, affordability of healthcare [81]
Social support	Strong social support networks [85, 93, 94] social difficulties [85] A culture that discourages the discussion of cancer or culturally appropriate cancer resources [93]
Financial support	Financial burden/ cost of care [89–91], limited funding [90, 97] Financial well-being [32]
Sex/intimacy	Impact of treatment/restriction/alteration in a sexual relationship and intimacy [14, 85, 89, 95, 97]
Coping/survival	Manage others' unhelpful beliefs, expectations, and emotions [84] Issues with survival and growth [84, 85] Barriers to employment during survivorship [94, 95] Approaches to post-treatment care (Infrequent clinical follow-ups, long distances to travel) [32]
Daily activity	Difficulties in performing household chores [86, 91], self-care activities, and shopping [86]
Spiritual support	Religion and spirituality [85, 90, 99]
Information support	Survivorship education and self-management [32, 85, 101] Lifestyle advice [32, 86, 90] Information about disease [88], side effects of treatment [98], and treatment plan [90] Available access to healthcare sources and choice of cancer specialists [37, 86, 90]
Medical counseling	Counseling [90, 91] Appropriate counselors [101]
Peers communication	Connecting with other survivors (patients) and caregivers [93, 101, 102]
Dignity	Dealing with self-concept change [84] Persistence of body image disturbance [88, 89, 94] Difficulty in disclose [90], and keeping their cancer a secret [85] Treatment with dignity and respect for a patient's opinion [90]

### Risk factors related to unmet needs

It was found that USCNs were significantly associated with many factors such as age, education, symptoms, treatment, stress, anxiety, and so on (Table 5), which could be summarized into three main aspects: demographic factors, disease factors, and psychological factors. Variables significantly associated with higher USCNs across all domains (psychological, health system and information, physical and daily living, patient care and support, and sexual) were indeterminate in age, marriage, occupational status, family income, level of education, and treatment time. The determinable single relationship was discovered in rural residents, short duration, combined treatment, advanced disease stage, poor performance status, higher depression, higher stress, higher distress, higher anxiety, poor QoL, symptoms severity, more comorbidity, and physical impairment.

**Table 5:** Risk factors related to USCNs

	**All domain**	**Sexuality**	**Information**	**Psychology**	**Physical/daily life**	**Patient care**
**Age**						
Young age	( +) [31, 40, 63, 64, 69, 73, 81]	( +) [51, 66]	( +) [66]			
Old age	( +) [61]		( +) [103]		( +) [71]	
**Marriage**						
Married	( +) [62, 81]	( +) [31, 62–64]				
Unmarried	( +) [40]				( +) [31, 63]	
**Occupation**						
Employed	( +) [31, 63, 73]					( +) [62]
Unemployed	( +) [51]		( +) [71]			
**Rural resident**	( +) [59]		( +) [59]			
**Short duration since diagnosis**	( +) [31, 51, 59, 63, 67, 73, 81]		( +) [67]	( +) [67, 76]	( +) [76]	
**Family income**						
Good	( +) [57]					
Poor	( +) [73]					
**Level of education**						
Low	( +) [59]		( +) [62]	( +) [66, 68]	( +) [66, 68]	
High	( +) [31, 40, 63]		( +) [32]	( +) [32]		( +) [66]
**Treatment time**						
Being under treatment	( +) [31, 74]	( +) [62]	(-) [75]			( +) [62]
Have completed treatment	( +) [62, 73]	( +) [103]	(-) [32]	( +) [32, 76]	( +) [66, 76]	( +) [75]
**Treatment method**						
Single				( +) [76]		( +) [62]
Combined	( +) [73]	( +) [103]	(-) [62]			( +) [62]
**Advanced disease stage**	( +) [31, 51, 59, 61, 63, 73]					
**Poor performance status**	( +) [51]			( +) [68]	( +) [68]	
**Higher depression**	( +) [42, 49, 67, 104, 105]			( +) [68, 105]	( +) [105]	
**Higher stress**	( +) [71]	( +) [56]				
**Higher distress**	( +) [42, 103, 106]		( +) [56]	( +) [56]	( +) [56]	
**Higher anxiety**	( +) [40, 42, 45, 49]		( +) [15]	( +) [105]		( +) [56]
**Poor QoL**	( +) [60, 67, 106]			( +) [61]	( +) [61]	( +) [61]
**Symptoms severity**	( +) [52, 69]	( +) [56]	( +) [56]	( +) [56]	( +) [56, 60]	( +) [56]
**Comorbidity**	( +) [49]				( +) [71, 103]	
**Physical impairment**	( +) [42, 73]					
**Others**	Level of survivorship concerns ( +) [104] Perception of illness ( +) [57] Family history of cancer ( +) [73] Social impairment ( +) [42]	Having children less than two ( +) [62]	Larger tumor size (> 2 cm) ( +) [66]	Relapse and terminal care patients ( +) [75]	The group with thoughts of suicide ( +) [71]	Invasive breast cancer ( +) [62]

( +) Positive correlation, (-) Negative correlation

## Discussion

From the cancer genomic revolution, and new inroads in immunotherapy for breast cancer to unique concerns of quality of life as well as survivors’ issues, these works represent much of the promise of breast cancer research as well as the challenges in the coming years [107]. There is a huge burden of supportive care needs among BCSs that are still under management, such as psychosocial issues [108], sexuality [109], information [110], and symptoms burden [111]. Most authors have investigated the USCNs among BCSs [112, 113] through cross-sectional study or qualitative interview. However, to our knowledge, few researchers conducted evidence synthesis [23, 114]. This scoping review aimed to explore the breadth and depth of existing literature on USCNs among BCSs, with the goal of obtaining an in-depth understanding of this topic. Overall, this scoping review identified 77 primary studies evidencing the USCNs of breast cancer survivors. The aims are trying to inform the prominent needs as well as influence factors, to provide guidelines for conveying superior cancer care.

### Quality appraisal

The results of the quality assessment of the involved research were presented in Figs. 2 and 3. The overall studies demonstrated a low to moderate risk of bias. It showed sufficient quality in terms of research method, data collection, and analysis. For quantitative research, there was an overall low risk of bias in sample size and appropriate sample frame. However, a high risk of bias was found in the detailed description of the study subjects and setting (44.2%), and how the participants were sampled (42%). The most used instrument was the self-made questionnaire and measurement heterogeneity were due to the use of unvalidated instruments. In the qualitative studies, the overall low risk of bias was found in conclusion drawing, ethical reporting, and representativeness of data. However, a high risk of bias was related to missing statements locating the researcher culturally or theoretically (79%), and the absence of stated philosophical perspective (54%).

### Assessment of USCNs

Many instruments are available to assess USCNs in breast cancer survivors. The most used instrument was the self-made questionnaire. Substantial heterogeneity was existing in their categories, development, and quality. The Short-form Supportive Care Needs Survey questionnaire (SCNS-SF34) was widely used in evaluating the need for supportive care among cancer patients with verified validity and reliability [115, 116]. However, the standardized assessment tools that are specific to people with breast cancer and their unique USCNs are absent. In our review, only Supportive Care Needs Survey-Breast Cancer (SCNS-Breast) [15] and Cancer Survivor Profile-Breast Cancer (CSP-BC) [73] were designed specifically for breast cancer patients. Meanwhile, few instruments covered all of the measurement properties [117]. Various unmet needs evaluation tools become problematic as domains assessed in our review often include psychological aspects, patient care and support, physical aspects and daily living, health system information, and sexuality [118], resulting in spiritual, social, and concerns for family or financial needs were under revealed. Besides, under most circumstances, the methodological quality was variable. In addition, dimension classifications of USCNs differ between instruments, which complicates comparisons within the literature. An urgent demand for a more specific instrument with universal applicability for BCSs should be emphasized. Meanwhile, qualitative research had provided some points that quantitative studies did not obtain. Compared to the fixed items, qualitative research provides a more flexible approach to expressing subjective experiences. Thus, the results of qualitative studies should serve as a meaningful reference for the construction and development of more specific evaluation tools.

### Prevalence of USCNs

Through making a comprehensive analysis of literature and summarizing them, 16 domains of USCNs were finally identified: physical/symptom need, psychological/emotional need, fear of cancer recurrence/ spreading, family support, medical support, social support, financial support, sexual/intimacy need, coping/survival need, daily activity need, spiritual support, information support, medical counseling, peer communication, cognitive needs, and dignity. This classification is more detailed, specific, and diversified than most previous studies [118–120], which could be helpful in clearly figuring out the definite unmet needs. In addition, extra USCNs were observed in concerns on caregiver burnout through qualitative studies, which indicated a need for appropriate support for their family/ caregiver/ partners. By estimating the pooled prevalence of USCNs from quantitative studies, it was found that social support (74%) counted the most proportion. However, with a small number of studies and large heterogeneity, caution must be applied as the findings might not be applicable to most breast cancer survivors. Even so, social support is still an indispensable part of BCSs. It was suggested that social support was significantly associated with resilience, posttraumatic growth [121], quality of life [122] and affective-cognitive symptoms [123]. Some social determinants such as poverty, lack of education, neighborhood disadvantage, racial discrimination, lack of social support, and social isolation were proven to significantly affect breast cancer incidence, stage at diagnosis, and survival [124]. In the present study, breast cancer patients commonly face unmet needs regarding social support in “help to handle the topic of cancer in social/work situation”, “a culture that discourages the discussion of cancer or culturally appropriate cancer resources”, “strong social support networks”, “social difficulties”. In the dignity domain, disease disclosure was also conveyed. It could be speculated that BCSs require adequate social support to in favor of their discussion and expression of the disease.

Daily activity (54%), sexual/intimacy (52%), fear of cancer recurrence/ spreading (50%), and information support (45%) were regarded as the top USCNs with high estimated prevalence. Information needs, psychological/emotional needs, physical/ symptom, medical support, and fear of cancer recurrence/spreading were been found to appear most frequently. In conclusion, fear of cancer recurrence/spreading and information need was the most reported with high pooled proportion and reporting frequency. Similarly, some previous studies have demonstrated that addressing recurrence concerns (80%) was the most commonly required [125]. Hypermutation occurs in 5% of all breast cancers with enrichment in metastatic tumors [126]. Fear of cancer recurrence (FCR) could be a powerful determinant of physical symptoms [127], psychological distress [128] and quality of life [129]. Our study demonstrated that BCSs not only faced the huge USCNs in FCR regarding “fears cancer spreading/recurrence”, but also in “dealing with the impact that having a faulty gene has had on your family”. It is not strange that the FCR is similarly reflected in the high information need related to hereditary disease. Psychological interventions might be an effective solution. A recent systematic review has recommended mindfulness and acceptance therapy-based interventions and short-term interventions to alleviate FCR [130]. Interventions to alleviate excessive worries and enhance feelings of personal control might help prevent or reduce related FCR [131].

Information needs were proved to be the most important concern among the diverse USCNs of cancer survivors [113]. Among BCSs, anxiety related to inadequate information support is common. A recent systematic review revealed that patients with breast cancer showed a huge enthusiasm in engaging intervention related to disease-focused information [132]. The prominent needs in the information domain vary among diverse patient groups. Patients with hematological malignancies were found to be mostly concerned about obtaining information about their future condition [9]. Meanwhile, more information about diet/nutrition in the form of a pamphlet or by a hospital dietician, and more information about the long-term self-management of symptoms and complications at home were discovered in patients with colon and/or rectum cancer [10]. A systematic review and synthesis of breast cancer patients' information needs developed a thorough information need model, including 3 themes, 19 categories, and 55 concepts [133]. In the present scoping review, “up to date understandable information about cancer and treatment”, “being informed about cancer which is under control or diminishing (i.e., remission)”, and “information related to hereditary disease” were the most stressed information need. Information needs regarding survivorship education, self-management, lifestyle advice and available access to healthcare sources, and choice of cancer specialists were also expressed. It inspired us to give more consideration in incorporating these unmet information needs into health education practice when delivering care for patients with breast cancer. It is believed information provision on BCSs could improve quality of life, reduce anxiety and increase intention to adhere to treatment recommendations [134]. American Society of Clinical Oncology Breast Cancer Survivorship Care Guideline has recommended that primary care clinicians should assess the information needs of breast cancer patients and its treatment, adverse effects, other health concerns, and available support services, and should provide or refer survivors to appropriate resources to meet these needs [135]. Technology-based or web-based seems to be an effective approach to provide enough information aid [136, 137]. Bootsma et al. integrated their investigation results about unmet information needs into a user-centered design to develop an informative website that targeted men with breast cancer [13].

Sexuality and intimacy represent a pillar of quality of life. The vast amount of evidence exists showing that cancer dramatically impacts a woman’s sexuality, sexual functioning, intimate relationships, and sense of self [138]. The overall prevalence of sexual dysfunction among female cancer survivors ranged from 16.7 to 67% [139]. Currently, sexual trouble is becoming more prevalent in BCSs owing to breast absence led by surgical treatment, body image, and adjuvant hormones. Low sexual desire persists throughout the timeline of BCSs, from BC diagnosis to after treatment [140]. Patients suffer from hot flashes, difficulty sleeping, loss of libido and intimacy, all resulting in significant morbidity and loss of quality of life [141]. The current finding exhibited that BCSs faced a majority of unsolved sexuality issues, particularly in diminished sexual activity/sexual drive, changes in sexual relationships and sexual feelings. A similar study conducted in gynecological cancer survivors revealed that they faced most sexual concerns on decreased sexual activity, emotional distancing from the partner, anxiety, and depression related to sexual performance [142, 143]. Among female cancer survivors, dyspareunia was the main type of sexual dysfunction reported after diagnosis [139]. Although, sexual issues are often neglected and not appropriately addressed by healthcare providers in their routine practice, which remains an unmet need with remarkable effects on general health and quality of life [144]. Effective communication between the health care professionals and cancer survivors was recommended to overcome this problem [139]. A review of the literature revealed trends utilizing psychoeducational interventions that include combined elements of cognitive and behavioral therapy with education and mindfulness training, which has positive effects on arousal, orgasm, satisfaction, overall well-being, and decreased depression [141].

### Factors associated with USCNs

Our present review showed that USCNs were significantly associated with demographic data, social determinants, disease status, quality of life, performance status, and some psychological indicators. However, causality cannot be determined due to the cross-sectional nature of the included studies. Meanwhile, due to the heterogeneity of research design, participation, and setting, a positive predictor in one article may be negative in another. Short duration since diagnosis, advanced disease stage, poor performance status, higher depression, higher stress, higher distress, anxiety, poor quality of life, more symptoms severity, existing comorbidity, and physical impairment, were identified to be significantly associated with higher USCNs of nearly all domains in most research. Compared to longer duration, a short duration since diagnosis might means more inadaptation no matter in physical or psychological or other aspects. As many studies had showed [70, 145, 146], high psychological issues, physical status, and poor quality of life were the strong predictive factors of high USCNs in BCSs. Patients who are assessed as high-risk need should be paid more attention in practice. Hence, the implementation of standardized screening tools in any phase of disease trajectory should be conducted for timely identification and intervention. In addition, prospective studies are needed to verify influencing factors that have a causal relationship with USCNs.

### Limitations and future directions

To the best of our knowledge, this study is the first and most comprehensive systematic scoping review regarding USCNs among breast cancer survivors. Firstly, through making a comprehensive analysis of literature and summarizing, a total of 16 domains of USCNs were finally identified. This classification is more detailed, specific, and diversified than most previous studies. Secondly, the most unmet supportive care needs were identified and the prominent needs lists of each domain were exhibited meticulously with proportion, through which the reader could obtain an in-depth understanding of USCNs among the breast cancer population. Thirdly, a comprehensive vision was provided to know potential influencing factors to USCNs for most of them were presented synthetically.

Even though, our study has some limitations. One of the limitations is the inclusion of literatures that are published only in English. In addition, there were amounts of studies without the full text. These may result in the exclusion of potentially useful research. What’s more, we failed to perform subgroup analysis because of the complexity and heterogeneity of the incorporated breast cancer population.

Research about USCNs among BCSs in more detailed classifications are needed to provide targeted supportive care, there is a need for more comparations among breast cancer patients in different subgroups. Also, an urgent demand for a more specific instrument with universal applicability for BCSs should be emphasized due to the heterogeneity of assessment tools. What’s more, we summarized the risk factors of unmet needs but failed to analyze the odds ratio (OR), hazard ratios (HRs), or relative risk (RR) of each variable. Data synthesis through meta-analysis or prospective study to determine the real factors are demanded.

## Conclusion

BCSs are experiencing the highest USCNs in fear of cancer recurrence, daily activity, sexual/intimacy, psychology, and information field. Various risk factors had been discovered to correlate with USCNs. Factors that have a causal relationship with USCNs should be identified through synthesizing longitudinal studies. There was substantial heterogeneity in study populations and assessment methods warranting future investigation considering specific samples and standard USCNs assessment tools that are validated for use in BCSs. Meanwhile, effective interventions based on guidelines should be formulated and conducted to decrease USCNs among BCSs in the future.

## Data Availability

All data relevant to the study are included in the article. All the raw data analyzed during this study could be obtained through contacting the first author.
